# Zika Virus-Like Particles Bearing a Covalent Dimer of Envelope Protein Protect Mice from Lethal Challenge

**DOI:** 10.1128/JVI.01415-20

**Published:** 2020-12-09

**Authors:** Giuditta De Lorenzo, Rapeepat Tandavanitj, Jennifer Doig, Chayanee Setthapramote, Monica Poggianella, Ricardo Sanchez-Velazquez, Hannah E. Scales, Julia M. Edgar, Alain Kohl, James Brewer, Oscar R. Burrone, Arvind H. Patel

**Affiliations:** a MRC—University of Glasgow Centre for Virus Research, Glasgow, Scotland, United Kingdom; b Molecular Immunology Group, International Centre for Genetic Engineering and Biotechnology, Trieste, Italy; c Department of Clinical Pathology, Faculty of Medicine Vajira Hospital, Navamindradhiraj University, Bangkok, Thailand; d University of Glasgow, Institute of Infection, Immunity and Inflammation, Glasgow, Scotland, United Kingdom; University of North Carolina at Chapel Hill

**Keywords:** vaccine, virus-like particles, Zika, neutralizing antibodies

## Abstract

Zika virus (ZIKV) envelope (E) protein is the major target of neutralizing antibodies in infected hosts and thus represents a candidate of interest for vaccine design. However, a major concern in the development of vaccines against ZIKV and the related dengue virus is the induction of cross-reactive poorly neutralizing antibodies that can cause antibody-dependent enhancement (ADE) of infection. This risk necessitates particular care in vaccine design. Specifically, the engineered immunogens should have their cross-reactive epitopes masked, and they should be optimized for eliciting virus-specific strongly neutralizing antibodies upon vaccination. Here, we developed ZIKV subunit- and virus-like particle (VLP)-based vaccines displaying E in its wild-type form or E locked in a covalently linked dimeric (cvD) conformation to enhance the exposure of E dimers to the immune system. Compared with their wild-type derivatives, cvD immunogens elicited antibodies with a higher capacity to neutralize virus infection in cultured cells. More importantly, these immunogens protected animals from lethal challenge with both the African and Asian lineages of ZIKV, impairing virus dissemination to brain and sexual organs. Moreover, the locked conformation of E reduced the exposure of epitopes recognized by cross-reactive antibodies and therefore showed a lower potential to induce ADE *in vitro*. Our data demonstrated a higher efficacy of the VLPs in comparison with that of the soluble dimer and support VLP-cvD as a promising ZIKV vaccine.

**IMPORTANCE** Infection with Zika virus (ZIKV) leads to the production by the host of antibodies that target the viral surface envelope (E) protein. A subset of these antibodies can inhibit virus infection, thus making E a suitable candidate for the development of vaccine against the virus. However, the anti-ZIKV E antibodies can cross-react with the E protein of the related dengue virus on account of the high level of similarity exhibited by the two viral proteins. Such a scenario may lead to severe dengue disease. Therefore, the design of a ZIKV vaccine requires particular care. Here, we tested two candidate vaccines containing a recombinant form of the ZIKV E protein that is forced in a covalently stable dimeric conformation (cvD). They were generated with an explicit aim to reduce the exposure of the cross-reactive epitopes. One vaccine is composed of a soluble form of the E protein (sE-cvD), the other is a more complex virus-like particle (VLP-cvD). We used the two candidate vaccines to immunize mice and later infected them with ZIKV. The animals produced a high level of inhibitory antibodies and were protected from the infection. The VLP-cvD was the most effective, and we believe it represents a promising ZIKV vaccine candidate.

## Abstract (precis)

Infection with Zika virus (ZIKV) leads to the production by the host of antibodies that target the viral surface envelope (E) protein. A subset of these antibodies can inhibit virus infection, thus making E a suitable candidate for the development of vaccine against the virus. However, the anti-ZIKV E antibodies can cross-react with the E protein of the related dengue virus on account of the high level of similarity exhibited by the two viral proteins. Such a scenario may lead to severe dengue disease. Therefore, the design of a ZIKV vaccine requires particular care. Here, we tested two candidate vaccines containing a recombinant form of the ZIKV E protein that is forced in a covalently stable dimeric conformation (cvD). They were generated with an explicit aim to reduce the exposure of the cross-reactive epitopes. One vaccine is composed of a soluble form of the E protein (sE-cvD), the other is a more complex virus-like particle (VLP-cvD). We used the two candidate vaccines to immunize mice and later infected them with ZIKV. The animals produced a high level of inhibitory antibodies and were protected from the infection. The VLP-cvD was the most effective, and we believe it represents a promising ZIKV vaccine candidate.

## INTRODUCTION

For decades, Zika virus (ZIKV) was largely ignored as a human pathogen, but the recent epidemic in South America has brought to light neurological complications (i.e., Guillain-Barré syndrome) (1) and congenital Zika syndrome (i.e., microcephaly and other malformations) (2), making ZIKV a public health threat in affected countries. ZIKV infection occurs mainly via mosquito bite, but its persistence in bodily fluids such as semen allows sexual transmission (3). There is currently no vaccine or treatment available, making their development a priority in ZIKV research.

Current approaches to vaccine development include purified inactivated virus (4), DNA/RNA/vector-based vaccines encoding structural proteins (5–10), and purified viral-like particles (VLPs) (11, 12) or protein subunits (13, 14). Some of these candidates are currently undergoing phase 1 clinical trials, but the design of a successful ZIKV vaccine is complicated by the close relation of ZIKV with other flaviviruses and especially dengue virus (DENV), also transmitted by *Aedes* mosquito vectors and overlapping among many areas.

The ZIKV genome, like that of other members of the *Flaviviridae* family, is composed of a positive-strand RNA encoding a single polyprotein that is cleaved into structural (capsid, precursor-membrane, and envelope) and nonstructural (NS1, NS2, NS3, NS4, and NS5) proteins. The envelope (E) glycoprotein, with its three domains (DI, DII, and DIII), is the main target of the host immune response (15). During the initial stages of flavivirus genesis, the E protein is associated with the precursor-membrane protein (prM) and assumes a trimeric conformation; only during passage through the trans-Golgi network, where the viral particle encounters an acidic environment, the trimers dissociate to reassemble as dimers (16). This new conformation is necessary to allow furin-mediated cleavage of prM into pr and M, generating a mature E dimer (17). Once released into the extracellular environment, pr dissociates and the particle becomes infectious. During infection, the low pH of the endosome triggers a new conformational modification that mediates fusion of viral and endosomal membranes (18). However, the particle maturation process is often incomplete, releasing a viral progeny partially displaying E protein in trimers containing prM. In addition, E protein is in continuous dynamic motion, a phenomenon called “virus breathing” that is strain- and temperature-dependent (19). These two factors—incomplete maturation and viral breathing—have important consequences on epitope accessibility. At its tip, DII harbors the fusion loop (FL), represented by an amino acid sequence that is highly conserved among flaviviruses. FL is masked by DI and DIII when E protein on the virion is in a dimeric form but becomes exposed upon rearrangement of E in the acidic endosome following cell entry. Epitopes located on DI/DII, especially in the FL region (FLE), are immunodominant but recognized by cross-reactive and poorly neutralizing antibodies (20, 21). This class of antibodies can be responsible for antibody-dependent enhancement (ADE) of infection, where antibody-bound virus particles are endocytosed via the Fcγ receptor, leading to a more severe infection (22). Antibodies to prM also contribute to ADE (22).

In addition, the most potent neutralizing antibodies often recognize complex quaternary epitopes that bind to multiple adjacent E proteins, epitopes that are available only when the E protein is assembled in a viral particle and therefore cannot be elicited upon immunization with subunits (23). Recently, a new class of quaternary epitopes, called the envelope dimer epitopes (EDE), has been described (24). EDE epitopes are displayed when the E proteins form a head-to-tail dimeric conformation. Highly neutralizing antibodies recognizing EDE were discovered in the sera of DENV-infected patients, but interestingly, they were also shown to efficiently neutralize ZIKV, both in *in vitro* and in *in vivo* experiments (25–27). Upon binding to prefusion E dimers, these antibodies can prevent the transition of E to a trimeric form and consequently abrogate membrane fusion and infection.

Here, we aimed to develop antigens for ZIKV vaccination that can drive the immune response preferentially against quaternary/complex epitopes to increase the neutralizing potential. Our recent study demonstrated that the introduction of a disulfide bridge by A264C substitution can stabilize E in a covalent dimer (cvD) conformation (28). This structure reduces the exposure of the unwanted FLE in favor of EDE. We generated cvD forms of a soluble E (sE-cvD) and a virus-like particle (VLP-cvD). The latter is expected to present E predominantly in the form of dimers, conferring a smooth surface to the particles. Vaccination of mice with these antigens afforded full protection from lethal ZIKV challenge. Moreover, in comparison to their WT counterparts, the cvD immunogens elicited antibodies that exhibited lower *in vitro* ADE of DENV, yellow fever virus (YFV), and West Nile virus (WNV). Our data confirmed the potential of cvD mutation in generating an immune response against neutralizing conformational epitopes and further identified VLP-cvD as the most promising candidate of the two cvD derivatives tested.

## RESULTS

### Design, expression, and purification of E covalent dimer-based vaccines.

We focused on designing antigens that would elicit antibodies to the complex quaternary epitopes that span two or more ZIKV E molecules. Immunogens based on EDE have great potential, but a stable dimeric conformation of E is not easy to achieve. For this reason, we used a strategy of generating a covalently stable dimeric form by introducing Ala to Cys mutation in DII (A264C) of E as described previously (28). The stable dimeric E generated is thus expected to enhance exposure of EDE and reduce presentation of the unwanted immunodominant FL region in DII to the immune system.

**FIG 1:**
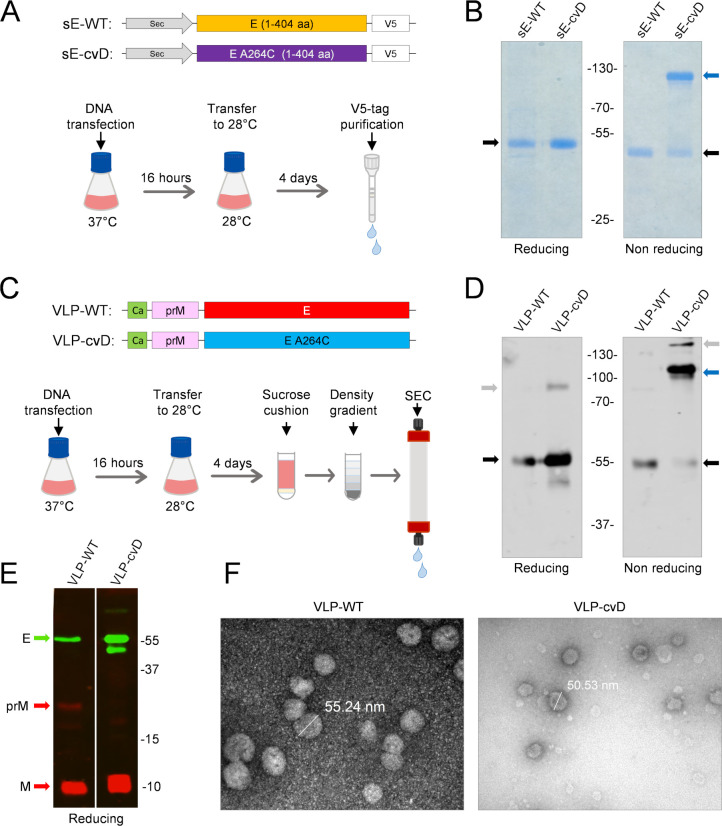
Expression, purification, and characterization of ZIKV E antigens. (A) Schematics of the genetic constructs used to express V5-tagged ZIKV sE (amino acids [aa] 1 to 404) in the WT or cvD (carrying A264C mutation) form. Expi293F cells were transiently transfected with the relevant constructs, and the expressed proteins secreted into the medium were purified using V5-tag affinity chromatography. (B) SDS-PAGE of the purified protein. sE-WT and sE-cvD were analyzed in SDS-PAGE run in the presence or absence of reducing conditions. Black arrows show monomers of sE, blue arrow shows dimers. (C) Schematics of VLP antigen design and purification: plasmid constructs carrying the sequences encoding the capsid anchor (i.e., the N-terminal 18 amino acids of capsid protein), followed by prM and full-length E genes, the latter in its WT form or in the form of cvD (i.e., carrying the A264C mutation). The constructs were transiently transfected in Expi293F cells, and the secreted VLPs were pelleted by sucrose cushion 4 days posttransfection and subsequently purified by density gradient followed by size exclusion chromatography (SEC). (D) Western Blot of the purified VLPs showing dimeric conformation: purified VLP-WT and VLP-cvD were analyzed by SDS-PAGE under reducing or nonreducing conditions. E was detected using an in-house made monoclonal antibody, DIII-1B. Black arrows show E monomers; blue arrow shows dimers; gray arrows show higher order oligomers (nonreducing gel) or partially resolved complexes (reducing gel) of the E protein. (E) Western Blot of the purified VLPs showing prM and M content: purified VLP-WT and VLP-cvD were analyzed by SDS-PAGE (14% acrylamide) under reducing conditions. Protein E was detected using the monoclonal antibody DIII-1B (in green), whereas proteins prM and M were detected using an anti-M antibody (in red). (F) Electron microscope pictures of purified VLPs: electron microscopy (uranyl acetate staining) of VLP-WT (left) and VLP-cvD (right) purified as described for panel C. Bars indicate the diameter of the particles.

We generated a V5 epitope-tagged soluble ZIKV E (sE; i.e., E lacking its stem and membrane anchor domains) in its wild-type form (sE-WT) and in the form of a covalently stabilized dimer (sE-cvD) containing the A264C mutation (Fig. 1A) (28). In addition, we also generated wild-type (WT) and cvD forms of ZIKV virus-like particles (VLPs) using plasmid constructs encoding the capsid anchor region (Ca) followed by the full-length prM and E (Fig. 1C). These proteins were produced by transient transfection of Expi293F cells with the relevant constructs and subsequently purified from the cell medium as described in Materials and Methods. The purified sE proteins were analyzed in SDS-PAGE gels under reducing and nonreducing conditions (Fig. 1B). As expected, the sE-WT was visible exclusively in the monomeric form, with an apparent molecular weight of ∼50 kDa, while sE-cvD under nonreducing conditions had an apparent molecular weight corresponding to a dimer (∼110 kDa) that was reduced to a monomer upon incubation with dithiothreitol (DTT). A small amount of sE-cvD was seen in a monomeric form under the nonreducing condition. VLPs were expressed in a similar fashion and purified as shown in Fig. 1C. SDS-PAGE and Western blot analysis confirmed the presence of dimeric E in VLP-cvD (∼110 kDa) when analyzed under nonreducing conditions, and this was reduced to a monomer in the presence of DTT (Fig. 1D). We also observed two additional minor bands: one in the nonreducing gel, where there was a higher molecular weight protein possibly representing a more complex aggregate of E, and an approximately 90-kDa protein in the reducing gel, which is likely an intermediate product resulting from an incomplete thiol reduction. In contrast, monomeric E (∼55 kDa) was found in the VLP-WT preparation under both reducing and nonreducing conditions. The molecular weight of E in the VLP preparations was higher than that of the two sE proteins (Fig. 1B) on account of the presence of the stem and anchor sequences. As expected, the viral M (10 kDa) was also detected in both forms of VLPs. Protein M is the product of furin-mediated cleavage of prM (25 kDa) during the maturation of virus particles. The presence of M protein in the absence of prM suggested that in VLP-cvD, the mutated glycoprotein underwent a complete maturation process during its synthesis, yielding smooth particles bearing the cvD E protein. Instead, the VLP-WT preparation contained residual prM, implying that they were not fully matured (Fig. 1E). Electron micrographs (Fig. 1F) of both types of VLPs showed particles of around 50 nm, comparable to the size of infectious ZIKV particles (29).

### Antibodies generated by cvD immunogens are conformation sensitive.

**FIG 2:**
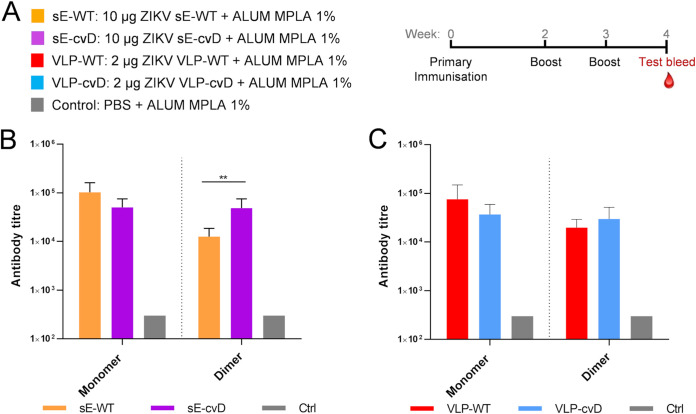
(A) Schematic representation of the immunization procedure: five groups, each (*n* = 6) of 4-week-old mice, received sE-WT, sE-cvD, VLP-WT, VLP-cvD, or PBS mixed with alum-MPLA adjuvant as shown. Following two boosts at weeks 2 and 3, test bleeds were collected at week 4 for analyses. Anti-E antibody titers of sera collected from animals immunized with sE (B) or VLP (C) proteins. Antibody titers were determined using ELISA plates coated with mono-biotinylated monomeric E and dimeric E. Ctrl, pooled sera from PBS control group. The titer was defined as the maximum dilution that gives a value higher than three-times the value given by the preimmune sera. The control sera were negative at the lowest dilution (1:900), and their titer was calculated as one-third of that dilution (300). Statistical analysis was performed using a 2-sided analysis of variance (ANOVA), 95% confidence level, with Tukey’s pairwise comparison at 95% confidence (Minitab software).

ZIKV cannot infect immunocompetent mice due to its inability to counteract the murine interferon response (30). We therefore used the interferon receptor-deficient transgenic knockout (*Ifnar1*^−/−^) A129 mice, which are susceptible to ZIKV infection and which have been shown to be amenable to vaccine evaluation studies (31). Cohorts, each of 4-week-old mixed male and female animals (*n* = 6), were vaccinated with sE-WT, sE-cvD, VLP-WT, VLP-cvD, or phosphate-buffered saline (PBS; as a control). Three doses of 10 μg (sEs) or 2 μg (VLPs) of protein adjuvanted with alum (1%) combined with monophosphoryl lipid A (MPLA) (5 μg) were administered by subcutaneous route as shown in Fig. 2A. One week after the last dose, blood samples were tested for the presence of anti-E antibodies.

We first tested the serum antibodies for binding to biotinylated derivatives of ZIKV sE proteins fused in-frame with the biotin-acceptor peptide (BAP). Specifically, recombinant sE-WT-BAP (monomer) or sE-cvD-BAP (dimer) coexpressed with the bacterial biotin ligase BirA (32, 33) (to allow *in vivo* mono-biotinylation) was used to quantify the titer of antibodies recognizing E in its monomeric or dimeric form.

A comparison of the binding levels showed that sE-cvD immunization elicited antibody titers against the dimer four times higher than those obtained with sE-WT. On the other hand, analysis of the sera from VLP-WT- and VLP-cvD-vaccinated animals by enzyme-linked immunosorbent assay (ELISA) showed no significant changes in the levels of antibodies (Fig. 2C). It should be noted that this ELISA format is not robust enough to discriminate antibodies binding to more-complex epitopes.

**FIG 3:**
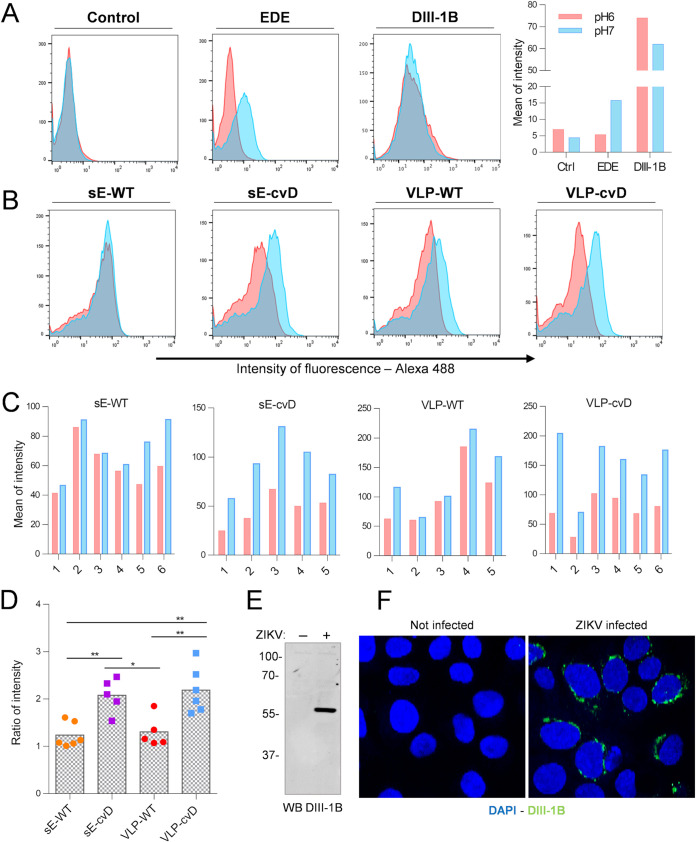
Determination of binding characteristics of serum IgGs to different sE conformations. (A) Cells expressing sE on the cell surface were incubated with pooled control sera (control), monoclonal EDE antibody 1C10 (EDE), or monoclonal DIII-1B antibody (DIII-1B) at pH 6.0 (red) or pH 7.0 (blue). Following washing, the bound antibodies were detected using a fluorescence-tagged secondary antibody, and the relative fluorescence was determined by flow cytometry using FACSCalibur. Mean of fluorescence intensity was calculated using FlowJo software and plotted (right). (B and C) Sera from immunized animals were incubated with cells as described for panel A. (B) Histogram plots show data from one serum sample as representative of each immunized group. (C) Bar charts showing data for sera from individual animals from each immunized group plotted as means of fluorescence intensity. (D) Ratios between the intensity of fluorescence at different pHs were calculated; gray columns represent means. Statistical analysis was performed using one-way ANOVA, 95% confidence level, with Tukey’s multiple comparison (GraphPad software). Data are representative of three independent experiments, performed with pooled and single serum samples. Characterization of the mouse monoclonal antibody (MAb) DIII-1B. MAb DIII-1B was obtained using standard hybridoma technology from BALB/c mice immunized with recombinant domain III of ZIKV E protein. (E) The specificity of MAb DIII-1B was tested by Western immunoblotting of VERO cells that were mock-infected (−) or infected with ZIKV (+). As expected, MAb DIII-1B specifically bound to ZIKV E protein. Protein molecular weight ladder is shown on the left (in kDa). (F) Separately, the binding specificity of MAb DIII-1B was also tested by indirect immunofluorescence of uninfected or ZIKV-infected A549-NPro cells. Green signal indicates antibody binding to ZIKV E protein. Cell nuclei were stained with 4′,6-diamidino-2-phenylindole (DAPI) (blue).

To further characterize the types of antibodies elicited by our cvD antigens, we used a recently developed cytofluorimetry assay of cells displaying dimers of ZIKV sE protein (M. Poggianella, G. Leccese, O. R. Burrone, and J. Slon-Campos, unpublished data). In this assay, the C terminus of sE is fused to the transmembrane and cytosolic tail of the type-I transmembrane protein major histocompatibility complex class I alpha (MHC-Iα) for plasma membrane display of the protein, as previously reported (28). This assay has the potential to discriminate antibodies binding exclusively to dimeric E on the basis of the pH-dependent mobility of E protein: at pH 7, which resembles the neutral extracellular environment, the protein is in a dimeric conformation, but at pH 6, mimicking the conformational changes that occur in the acidic endosome vesicles during infection, it moves to a prefusion monomeric conformation. When exposed to a neutral pH (pH 7), E can physiologically dimerize and therefore be recognized by the dimer-specific monoclonal antibody EDE 1C10 (24). However, this interaction is completely abrogated when cells are exposed to a lower pH (pH 6), due to the disruption of the dimer. Thus, with this dimer-specific antibody, two populations of cells can be detected by flow cytometry—antibody bound and unbound—depending on the assay conditions (Fig. 3A, EDE). On the other hand, antibodies binding to epitopes that do not require dimeric conformation of the protein are poorly affected by the pH and therefore show no differences in the binding capacity, as shown using in-house made monoclonal antibody DIII-1B (Fig. 3A, DIII-1B), which recognizes a linear epitope located on domain III (Fig. 3E and F). This assay is primarily designed, and indeed works optimally, for monoclonal antibodies. Nevertheless, we reasoned that it would still be useful in evaluating the nature of antibodies in sera from vaccinees containing a mix of IgGs capable of binding to linear or conformational epitopes. As shown in Fig. 3B to D, serum antibodies from sE-WT- and VLP-WT-immunized groups seemed to not be particularly affected in the binding by the pH-dependent change of conformation. In contrast, sera from sE-cvD- and VLP-cvD-vaccinated animals showed a more consistent pH-dependent difference in the relative peak positions (Fig. 3B to D).

Taken together, the data suggest that cvD antigens elicited a population of antibodies more sensitive to changes in conformation then the one elicited by the WT immunization.

### cvD antigens elicit neutralizing antibodies in mice.

**FIG 4:**
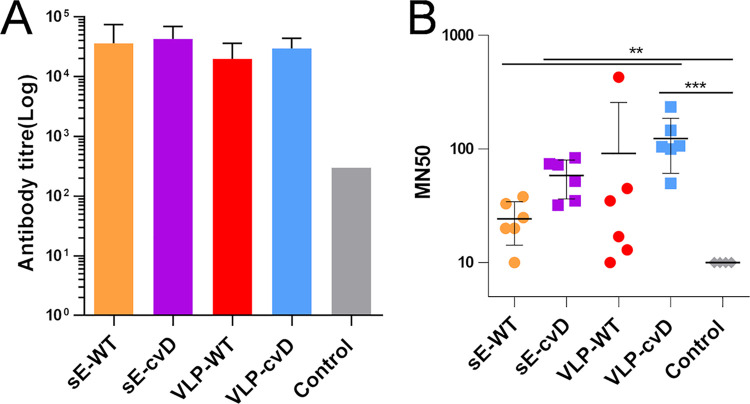
Determination of anti-E or neutralizing antibody titers for sera from vaccinated animals. (A) Anti-E titers: ELISA plates coated with biotinylated dimeric E were incubated with serially diluted serum samples, and the bound antibodies detected as described in Materials and Methods. Antibody titers were determined as described in the legend for Fig. 2. (B) Neutralization of ZIKV infection: serially diluted samples of mouse sera were incubated with ZIKV for 1 h before infecting Vero cells. At 72 h postinfection, the intracellular levels of E were determined by capture sandwich ELISA, and the percentage infectivity relative to that of the virus alone was calculated. The results were plotted as MN_50_ values, i.e., titers at which 50% neutralization was achieved. Statistical analysis was performed using 2-sided ANOVA, 95% confidence level, with Tukey’s pairwise comparison at 95% confidence (Minitab software). Data from three independent experiments were used.

A new immunization was performed using the same procedure shown in Fig. 2A. Antibody titers were measured using exclusively the sE-cvD-BAP ELISA (Fig. 4A). The neutralizing capacity of these sera was then determined in a micro-neutralization (MN) assay that we had previously developed (9). This sandwich ELISA accurately measures the levels of glycoprotein E in infected cells, thus enabling quantitation of virus infectivity. Vero cells were infected with the Puerto Rican ZIKV strain PRVABC59 (an Asian lineage isolate) that had been preincubated for 1 h with serially diluted mouse sera. Three days postinfection, the level of cellular E protein was determined by sandwich ELISA. Percentage of infectivity was calculated relative to E yield in cells infected in the absence of sera. As shown in Fig. 4B, antibodies elicited by sE-cvD and VLP-cvD significantly neutralized virus infection compared to sera from the control group. VLP-cvD sera neutralized more strongly than sE-WT sera. Although not statistically significant, we observed a trend toward higher *in vitro* neutralization titers from cvD-elicited sera than from their WT counterparts (Fig. 4B).

### cvD immunogens protect mice from ZIKV challenge.

**FIG 5:**
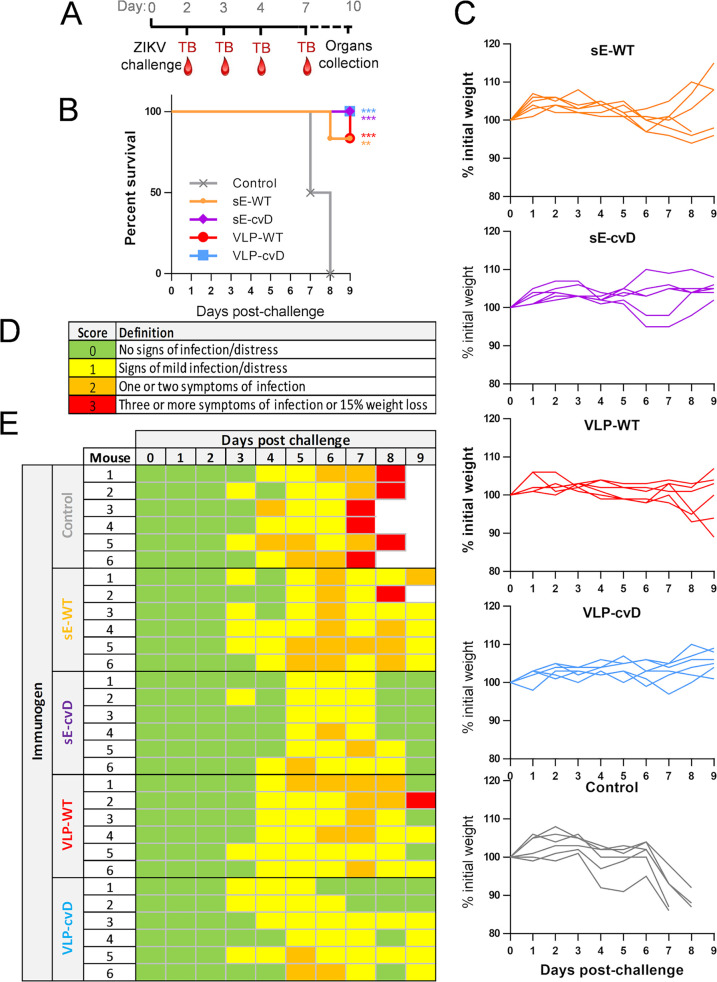
*In vivo* efficacy of candidate vaccines. (A) Schematic representation of the *in vivo* challenge protocol: mice were challenged with 10^4^ PFU of ZIKV PRVABC59 1 month after the primary immunization and were monitored for up to 9 days. Test bleeds (TB) and organs were collected as shown. Animals were weighed (C) and scored for clinical signs daily postchallenge, with percentage of survival shown in panel B. (D) Legend of scoring system used to monitor animal health following ZIKV challenge. (E) Table showing the score attributed to each animal after ZIKV PRVABC59 challenge. Animals displaying a weight loss of 15% or more were euthanized. All the members of the control group reached the endpoint score 7 to 8 days postchallenge and were therefore euthanized. Statistical analysis was performed using log rank (Mantel-Cox test) with GraphPad Prism software.

**FIG 6:**
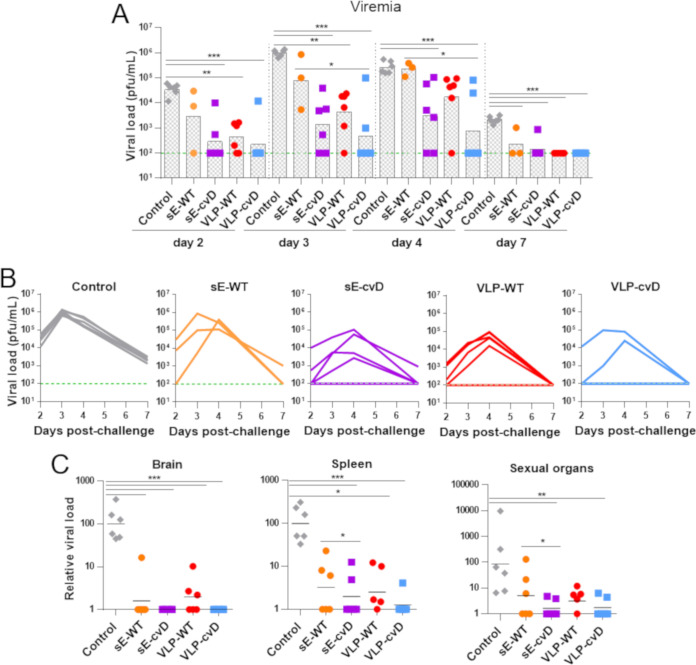
Viral titer in challenged animals. (A) The levels of ZIKV in the serum at day 2, 3, 4, and 7 postinfection were quantified by RT-qPCR and the results plotted as PFU per milliliter. (B) The limit of detection was estimated to be 100 PFU/ml, indicated by the green line. Columns show means from all mice. (C) Relative viral loads in brain, spleen, and sexual organs: the presence of viral RNA in tissues was quantified by RT-qPCR, and the results are plotted as relative viral load calculated using the average from the PBS control group. Statistical significance is reported. Statistical analysis was performed using two-sided ANOVA, 95% confidence level, with Tukey’s pairwise comparison at 95% confidence with Minitab software.

To assess *in vivo* efficacy of our candidate vaccines, we challenged the above-described immunized animals with 10^4^ PFU of ZIKV PRVABC59 by subcutaneous injection, as shown in Fig. 5A. A scoring system was used to monitor the progress of the disease based on the severity of clinical signs and symptoms, as described in Fig. 5D. A score of 3 was considered the humane endpoint. Animals were monitored for 9 days for their body weight changes (Fig. 5C) and clinical signs (Fig. 5E). The PBS control group began losing weight at 4 days postchallenge (dpc) and subsequently exhibited clinical signs of infection and were euthanized at 7 to 8 dpc (Fig. 5B and C, gray lines). The sE-WT group lost less weight but exhibited clinical signs comparable to those seen in the PBS control group albeit with delayed kinetics (Fig. 5B and C, orange lines). One mouse of the sE-WT group succumbed to infection. Similar profiles of weight change, clinical scores, and survival were observed in the VLP-WT group (Fig. 5B and C, red lines) compared to those in the sE-WT group. Importantly, all animals immunized with cvD antigens survived the challenge, maintained a more stable weight profile, and showed rapid recovery from the clinical signs of infection (Fig. 5B and C, purple and blue lines). Viremia was determined by reverse transcription-quantitative PCR (RT-qPCR) on blood samples taken at days 2, 3, 4, and 7 during the course of the challenge (Fig. 6A and B). Since the limit of the assay was determined as a titer of 10^2^ PFU equivalents/ml, for statistical analysis, this value was given to all the samples that were below the limit of detection. As expected, PBS control mice showed very high viremia (>10^6^ PFU/ml), which peaked at 3 dpc. In contrast, in all vaccinated animals, the viremia peaked at 4 dpc, although the levels varied. Specifically, the sE-WT-vaccinated animals displayed levels comparable to those observed in the PBS control group (>10^5^ PFU equivalents/ml). Instead, consistent reduction in viremia levels was observed in VLP-WT-, sE-cvD-, and VLP-cvD-vaccinated animals which, in the latter two groups, was significant. In particular, the geometric mean of viral titer of 4 × 10^2^ PFU equivalents/ml was the lowest in VLP-cvD-vaccinated group. Relative organ viral load was analyzed by RT-qPCR of viral RNA extracted from brain, spleen, and sex organs, which were collected immediately after euthanasia (Fig. 6C). The comparative threshold cycle (ΔΔ*C_T_*) method was used to calculate the titer relative to an average from the PBS control group. Although all four antigens reduced brain viral load, only cvD antigens reduced that of the sex organs. In the case of spleen viral transmission, sE-cvD and VLP-cvD were better than the sE-WT and control groups, whereas VLP-WT was only better than the control group. All together, these data confirmed the unsuitability of wild-type antigens (especially sE-WT), whereas both cvD derivatives conferred full protection against ZIKV infection *in vivo*.

### cvD reduces *in vitro* ADE.

**FIG 7:**
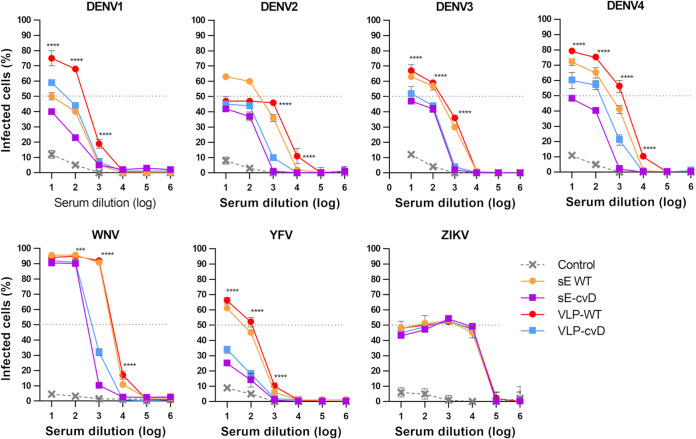
Effect of pooled sera on ADE of infection by all four DENV serotypes (1 to 4), ZIKV, WNV, and YFV. Viruses were preincubated with 10-fold dilution of pooled sera for 1 h before infecting K562 cells. Percentage of infected cells was calculated by cytofluorimetry. Control consists of pooled sera from PBS-injected mice. Statistical significance applies to the comparison between VLP-WT and sE-cvD/VLP-cvD. Statistical analysis was performed using two-sided ANOVA, 95% confidence level, with Tukey’s pairwise multiple comparison (GraphPad software). Experiments were performed in triplicates.

Due to the close relationship with DENV and other mosquito-borne flaviviruses, a ZIKV vaccine is very likely to elicit cross-reactive antibodies that may fail in neutralizing other flavivirus infection and instead lead to a worse disease outcome. Our candidate vaccines are designed to reduce this risk, limiting exposure of highly cross-reactive but low cross-neutralizing epitopes in favor of broadly neutralizing ones. We performed *in vitro* ADE assays using the K562 monocyte cell line that expresses the Fcγ receptor. Infection of these cells does not occur through the normal entry pathway. Rather, it occurs exclusively via Fcγ receptor-mediated internalization of the antibody-bound virus. In keeping with this, we observed an extremely low infection rate in the absence of antibodies binding to the virus, i.e., when the virus was preincubated in the absence of serum (data not shown) or in the presence of the sera from our PBS-injected mice. Viruses preincubated with 10-fold serial dilutions of the sera were added to the cells, incubated for 3 days, and then analyzed to determine the percentage of infection by cytofluorimetry. The experiment was performed in triplicates (Fig. 7).

We used ZIKV as a control in the assay. As expected, preincubation with all the sera gave the same pattern of infection, suggesting that antibodies (neutralizing or otherwise) in the four groups of sera bind to ZIKV similarly to mediate infection. This is not surprising, since internalization occurs independently of the normal receptors; therefore, the neutralizing antibodies are no longer capable of blocking entry. When tested against the four DENV serotypes, YFV, and WNV, sera from the sE-WT immunized group conferred infection of between 50% and 100% of cells. Instead, 10 times lower levels of infection were observed with sE-cvD sera, suggesting the presence of a much lower level of cross-reacting antibodies in these sera. Similarly, sera from VLP-cvD-immunized mice exhibited a 10-fold reduced infectivity compared to that with sera from VLP-WT animals. Particularly interesting was the level of infected cells obtained after virus incubation with VLP-WT sera, which was significantly higher than the infection obtained following incubation of the virus with the sera from sE-cvD- and VLP-cvD-immunized animals. These results suggest that the covalent dimer-based E vaccines (both sE-cvD and VLP-cvD) confer a lower risk of ADE than their WT counterparts as determined by this experimental model.

### VLP-cvD protection coverage includes ZIKV African lineage.

**FIG 8:**
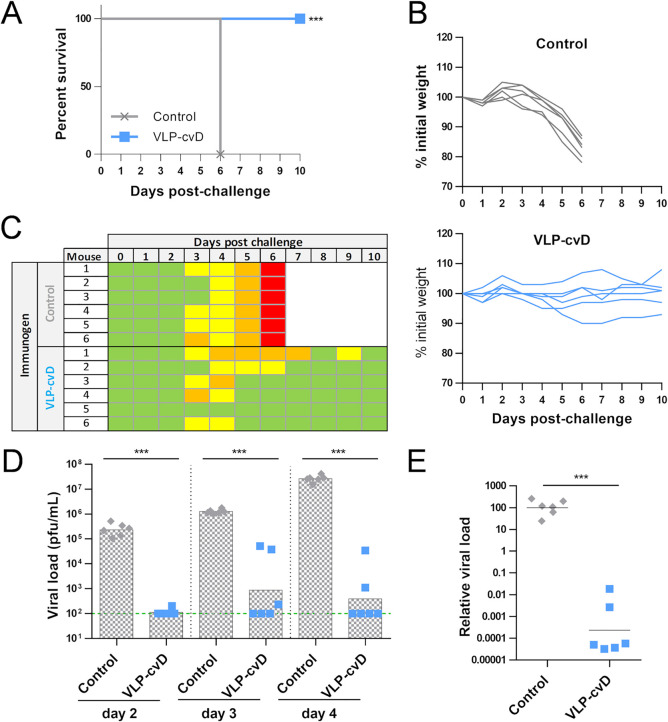
(A) Survival rate of vaccinated animals upon ZIKV challenge: mice were challenged with 10^4^ PFU of ZIKV MP1751. All the members of the control group reached the endpoint score 6 days postchallenge and were therefore euthanized. Statistical analysis was performed using log rank (Mantel-Cox test) with GraphPad Prism software. (B) Body weight variations after challenge: weight loss of mice after ZIKV infection. Animals showing a weight loss of 15% or higher were euthanized. (C) Clinical scores attributed to each animal after ZIKV MP1751 challenge. (D) Viral load in serum: the presence of ZIKV in the serum at days 2, 3, and 4 postinfection was quantified by RT-qPCR. Green line indicates the limit of detection. (E) Relative viral load in brain: presence of viral RNA in tissues was quantified by RT-qPCR. Statistical analysis was performed using two-sided two-sample *t* test, 95% confidence level, with Minitab software.

ZIKV diverged decades ago into two lineages, the African and the Asian. Whereas the Asian lineage is responsible for the last epidemics and is linked to neurological outcomes and birth defects, the African lineage is—intriguingly—well known to be more pathogenic in *in vivo* models (34). Since a safe ZIKV vaccine should guarantee coverage of both African and Asian lineages, we tested the VLP-cvD protectivity upon infection with a Ugandan (MP1751) isolate of ZIKV. Immunization and challenge were performed as previously described (Fig. 2A and 5A). The PBS group lost weight starting from day 3 postchallenge, and all mice reached the endpoint at day 6 (Fig. 8A and B, gray lines, and Fig. 8C). The VLP-cvD-vaccinated group instead showed a stable body weight, and all animals survived the challenge (Fig. 8A and B, blue lines, and Fig. 8C). PBS-immunized control mice showed high peak of viremia (>10^7^ PFU/ml) at 4 dpc, while the vaccinated mice showed a highly significant reduction in viremia (∼10^2^ PFU/ml) (Fig. 8D). Also, virus dissemination to the brain was suppressed in the VLP-cvD-immunized mice (Fig. 8E). All together, these results confirm the broadly protective potential of our vaccine candidate.

## DISCUSSION

Monoclonal antibodies recognizing quaternary epitopes that span more than one E protein are reported to be the most neutralizing and cross-reactive class of antibodies (24, 35, 36). Nevertheless, they constitute only a minority of the antibodies elicited by a natural infection, where the larger response focuses on poorly neutralizing and cross-reactive epitopes located on DI/DII (15, 37). The incomplete maturation of particles and the mobility of E reduce their exposure, especially to dimeric epitopes, in favor of the fusion-loop epitope. However, our vaccines are designed to lock E in a dimeric conformation, impairing its disassembly and forcing the protein to display the desired epitopes.

E homodimer stability was proved several times to be affected by temperature: dimerization is favored at 28°C and reduced at 37°C; hence, physiological temperature is another element that impairs dimer exposure to the immune system (38). But 28°C is also a crucial temperature for expression and secretion of E protein, both in the soluble form and as part of VLPs (28). Since this temperature is not compatible with genetic vaccination approaches, in which DNA or RNA encoding the antigen is administered and the antigen is then produced by the host cells at physiological temperature, the full potential of our antigens as candidate vaccines was evaluated in a protein-based vaccination approach.

Recently, a characterization of two dimeric E antigens was published, in which dimerization was achieved by the A264C mutation or by replacing the E transmembrane domain with the FC fragment of a human IgG (39). While the manuscript was under preparation, two other articles reported the development and evaluation of dimer-based subunit vaccines similar to that described here (40, 41). The authors showed that in all three cases, the antigens were able to induce in mice the production of neutralizing antibodies. However, our work is the first application of E covalent dimers to virus-like particles that proved, in our hands, to be a vaccine candidate superior to the soluble dimer.

We tested the potential of E covalent dimers when expressed in the form of soluble protein, lacking the stem anchor, and also as VLPs. In comparison with that for sE-WT, we observed a dramatic effect of the cvD mutation on the immune response, with a drastic reduction of antibodies binding to monomeric E and an impressive increase in protective activity upon lethal challenge. When the comparison was performed with the VLPs, the effect was less dramatic in terms of anti-dimer antibody titers. This is likely due to the thermal stability of dimers that is higher in ZIKV than in DENV (42). ZIKV E dimers are more stable, as already proven by the necessity of a double disulfide bridge to lock the DENV E dimer when one bridge is sufficient in ZIKV E. In addition, our ELISA and binding assays are based on E subunits presented in a monomeric or dimeric form but cannot fully recreate the complex symmetry of E protein on the viral particles and therefore cannot quantify the contribution of antibodies binding to adjacent dimers. However, the difference in antibody response and protectivity was enough to achieve an important reduction in DENV ADE. In this regard it is noteworthy that the VLP-cvD lacked prM, indicating their complete maturation. In contrast, VLP-WT contained prM that can elicit anti-prM antibodies upon vaccination. The prM protein is naturally present in DENV or ZIKV particles, due to incomplete maturation, but prM antibodies from DENV patients showed no or poor neutralizing activity and may instead likely induce ADE (43). All together, these observations strongly suggest that covalently linked E dimer can bring even greater benefits to the development of a DENV vaccine.

The development of a ZIKV vaccine requires attention to the possible cross-reaction with DENV. ADE of infection between different DENV serotypes is widely recognized as the cause of dengue shock syndrome (DSS), but the role that ZIKV infection may play at any point in influencing DENV infections is still unclear. Unfortunately, it is not easy to experimentally predict ADE, since results obtained from *in vitro* and *in vivo* settings and those from among different *in vivo* models are not always consistent. So far, animal models have not been able to replicate the full repertoire of the antibody responses in humans. In addition, it is difficult to reproduce severe DENV infection in animal models; in most cases, the severity of infection is based on increased viremia in the infected animals. *In vitro* tests performed with DENV-positive sera or DENV monoclonal antibodies showed cross-reactivity and ADE of ZIKV infection, similar to experiments performed in mouse models (20, 44–46). Conversely, analysis performed in nonhuman primate models ruled out a negative effect of previous DENV exposure on ZIKV infection (47), which was later confirmed by population studies with asymptomatic ZIKV infection in subjects positive for DENV antibodies (48). Concerns of cross-reaction and ADE were also raised about vaccination against other flaviviruses. *In vitro* studies supported negligible risk of ADE of ZIKV after tick-borne encephalitis virus vaccination, and no clinical evidence of increased disease severity in vaccinated people has emerged so far (49). The fear of predisposing a vaccinated individual to DSS generated a reluctance to deploy the YFV vaccine in DENV areas of endemicity, but a recent long-term study showed no evidence of increased risk (50). Not much is known about the role of WNV antibodies in causing other flavivirus ADE and vice versa. Like that with DENV, sera from WNV patients exhibit *in vitro* ADE of ZIKV (45). However, while DENV ADE is a well-documented phenomenon, to our knowledge, there is as yet no evidence of ADE occurring between DENV/ZIKV and WNV in humans.

However, the sequence homology between ZIKV and DENV is high, with elevated antibody cross-reactivity (15, 20). According to a recent publication, ZIKV infection can increase risk of a severe DENV2 disease to the same level as a previous heterologous dengue infection (51); therefore, how ZIKV vaccination can affect DENV pathogenesis is a pertinent question that remains to be addressed.

We evaluated the ADE potential (on DENV, YFV, and WNV) of the different sera using K562 cells. These cells express high levels of FcγRIIA and significantly favor FcγR-mediated infection, making them suitable for studying ADE in an *in vitro* setting. We found that sera from sE-WT-immunized animals exhibited high ADE in our assay, presumably because the antigen exposes well-known conserved epitopes, such as the FLE, allowing a high level of cross-reactivity. In contrast, ADE was strongly reduced when E was locked in the dimeric form (sE-cvD) and properly folded, likely because conserved but poorly neutralizing epitopes on DI/DII are not exposed. We obtained interesting results upon comparison of sera from mice immunized with VLPs bearing a WT or a cvD form of the full-length ZIKV E glycoprotein. While the VLP-WT conferred protection from lethal infection in mice, the sera from the same immunized animals induced *in vitro* ADE of ZIKV at levels comparable to that for sE-WT-immunized sera or even higher, as in the case of DENV1 and DENV4. In contrast, and as with sE-cvD, sera from VLP-cvD-immunized animals exhibited strongly reduced ADE of the flavivirus under analysis. This raises safety concerns about a vaccine that, despite protecting from ZIKV infection, may bring more adverse effects on subsequent DENV infections. Once again, this risk is reduced with the VLP-cvD antigen. However, to what extent ADE *in vitro* mimics any *in vivo* effects remains to be determined.

ADE of DENV upon ZIKV immunization is a risk that cannot be underestimated. One way to reduce antibody cross-reactivity would be to focus on developing a vaccine with a less-conserved subunit, such as DIII. However, we recently showed that this approach fails in protecting from *in vivo* challenges (52). The development of an engineered safe vaccine is probably an essential requirement to tackle this concerning public health challenge. Our two immunogens proved the high potential of engineered E protein locked in a dimeric conformation as a suitable vaccine candidate, with the most promising results achieved when the protein is part of a structurally more complex antigen presented in the form of a virus-like particle.

## MATERIALS AND METHODS

### Cell lines and virus strains.

Expi293F (Thermo Fisher Scientific) embryonic human kidney cells adapted to serum-free conditions were grown in Expi293 Expression medium as per the manufacturers’ protocol. Vero E6 cells were grown in Dulbecco’s modified Eagle’s medium (DMEM) (Life Technologies) containing 10% fetal bovine serum (FBS) (Life Technologies) and penicillin-streptomycin (Gibco). K562 cells were grown in RPMI 1640 medium (Life Technologies) containing 10% fetal bovine serum (FBS) (Life Technologies). ZIKV PRVABC59 (kindly supplied by BEI Resources; accession number KX087101) and ZIKV MP1751 (005V-02871; kindly supplied by Public Health England; accession number KY288905.1) were used for infection experiments, micro-neutralization, and animal challenge.

### Plasmid DNA constructs.

The ZIKV sE-encoding sequence (codons 1 to 404) was amplified from ArD158095 strain (accession number KF383121.1) as described by Slon Campos et al. (28). sE fused to an N-terminal immunoglobulin leader sequence (sec) and a C-terminal V5 tag (GKPIPNPLLFLD) was cloned into a pVax vector. A mammalian codon-optimized ZIKV prME gene sequence, flanked by the C-terminal portion of C and the N-terminal reside of NS1, was obtained from the ZIKV PE243 Brazilian strain (accession number KX197192.1 [53]) (amino acids [aa] 105 to 815 of the polyprotein) and cloned into a pDIs vector. The A264C mutation was introduced by site-directed mutagenesis into both plasmids.

### Protein expression and purification.

sE-WT, sE-cvD, VLP-WT, and VLP-cvD were expressed using an ExpiFectamine 293 transfection kit (Thermo Fisher Scientific) according to the manufacturer’s instructions. After 16 h, cells were moved to 28°C. At 5 days posttransfection, the supernatant was harvested and filtered. sE proteins were purified using the V5-tagged protein purification gel (Caltag Medsystems Ltd.), eluting with 2 mg/ml of V5 peptide. VLPs were pelleted by ultracentrifugation (115,000 × *g*, 4°C, 2 h) (Sorvall discovery 90SE with Surespin630 rotor) through a cushion of 20% sucrose in TN buffer (20 mM Tris and 120 mM NaCl). The pellet was resuspended in TN buffer and loaded on a discontinuous density gradient made by sodium potassium tartrate and glycerol in TN buffer (29). Tartrate concentrations ranged from 10% to 30%, with an interval of 5%, while that of glycerol ranged from 7.5% to 22.5%, with an interval of 3.75%. After centrifugation (Sorvall discovery 90SE with TH641 rotor) at 175,000 × *g* at 4°C for 2 h, fractions were collected and analyzed for the presence of ZIKV E by Western blotting. ZIKV E protein-positive fractions were pooled, dialyzed against Dulbecco’s phosphate-buffered saline (DPBS) (Life Technologies), and concentrated using a spin column (Amicon Ultra-15 [100 kDa]; Merck Millipore) before being subjected to size exclusion chromatography. Briefly, ∼500 μl of concentrated pooled fractions was loaded onto HiPrep 16/60 Sephacryl S-500 HR columns (GE Healthcare), and then 1.5 column volumes of mobile phase (DPBS) was run through the column at flow rate of 0.5 ml/min using the AKTA Pure (GE Healthcare) system. Fractions were collected and tested for ZIKV E protein. Positive fractions were pooled and concentrated using the Amicon Ultra-15 (100 kDa; Merck Millipore) spin column. The concentration of the purified proteins was determined using a NanoDrop One (Thermo Scientific).

### SDS-PAGE and Western blotting.

sE samples were subjected to 10% SDS-PAGE, and the fractionated proteins were detected by direct staining of the gel with InstantBlue (Sigma) or by Western blotting. VLP samples were separated by 10% or 14% SDS-PAGE, blotted to a polyvinylidene difluoride (PVDF) membrane (Immobilon-FL; Merck Millipore), blocked overnight with ODYSSEY blocking buffer (LI-COR), and then incubated with DIII1B antibody (anti-ZIKV E DIII generated in-house as described in Fig. 3) or ZIKA prM antibody (GeneTex) for 1 h. This was followed by incubations with anti-mouse IgG (IRDye 800CW; LI-COR) and anti-rabbit IgG (IRDye 680RD; LI-COR). Images were acquired by a LICOR machine.

### Electron microscopy.

VLPs were adsorbed for 3 min to Formvar carbon films mounted on 400-mesh per inch copper grids (Agar Scientific). Samples were washed three times with distilled water and stained with 2% saturated uranylacetate (Agar Scientific) for 2 min at room temperature. Specimens were analyzed in a transmission electron microscope (JEM-1200 EX II; JEOL) equipped with a charge-coupled-device (CCD) camera (Orius, Gatan) at an acceleration voltage of 80 kV.

### Animal immunization.

Four-week-old male and female *Ifnar1*^−/−^ mice (A129, 129S7 background; Marshall BioResources) (*n* = 6) were subcutaneously immunized with ZIKV antigen formulated in aluminum hydroxide gel (1% alum; Brenntag) combined with 5 μg monophosphoryl lipid A (MPLA) (InvivoGen) or PBS containing the adjuvant. Purified sE antigens used in each immunization contained 10 μg protein, while it was 2 μg in the case of VLPs. Mice were immunized at 0, 2, and 3 weeks and bled 4 weeks after primary immunization for antibody titration and the micro-neutralization assay. Four weeks after primary immunization, mice were challenged subcutaneously with 10^4^ PFU of Puerto Rican ZIKV (PRVABC59) or Uganda ZIKV (MP1751). Blood was collected at 2, 3, 4, and 7 dpc, and 10 μl of sera was assessed by RT-qPCR. Mice were euthanized when they exhibited three or more signs of moderate severity or lost more than 15% body weight; otherwise, they were euthanized 9 or 10 days after challenge.

### Animal ethics.

All animal research described in this study was approved by the University of Glasgow Animal Welfare and Ethical Board and was carried out under United Kingdom Home Office Licenses, P9722FD8E, in accordance with the approved guidelines and under the UK Home Office Animals (Scientific Procedures) Act 1986 (ASPA).

### ELISA.

Recombinant biotinylated proteins (sE, sE-cvD, and DIII) were expressed at 28°C using an ExpiFectamine 293 transfection kit (Thermo Fisher Scientific). Cell supernatant was harvested and dialyzed. Biotinylated proteins were captured in ELISA plates precoated with 5 μg/ml of avidin (Sigma) in Na_2_CO_3_-NaHCO_3_ buffer (pH 9.6) and subsequently blocked with PBS containing 0.05% Tween 20 and 1% bovine serum albumin (BSA; Sigma). Serial dilutions of mouse sera were tested for binding to the biotinylated proteins, and the bound antibodies were detected using horseradish peroxidase (HRP)-conjugated anti-mouse IgG A4416 (Sigma) and 3,3',5,5'-tetramethylbenzidine (TMB) substrate (Life Technologies).

### Antibody binding assay.

HEK cells stably expressing ZIKV sE protein on the surface were blocked in 1% BSA in PBS at pH 6 or 7 and then incubated with mouse sera diluted 1:500 in the same solution. After washing, cells were incubated with secondary anti-mouse Alexa Fluor 488 (Jackson ImmunoResearch) at 1:50,000 in 1% BSA PBS (pH 7) and analyzed by cytofluorimetry in a FACSCalibur (BD Biosciences) instrument.

### Micro-neutralization assay.

This assay was performed as described by Lopez-Camacho et al. (9). Briefly, 7 × 10^3^/well of Vero cells were seeded in 96-well plates and incubated at 37°C in 5% CO_2_. The next day, 3-fold serially diluted mice sera were first incubated at 37°C for 1 h with 100 PFU/well ZIKV strain PRVABC59. The serum/virus mix was then used to infect cells. After 1 h of incubation at 37°C, 100 μl of medium was added to each well. At day 3 postinfection, cells were lysed in lysis buffer (20 mM Tris-HCl [pH 7.4], 20 mM iodoacetamide, 150 mM NaCl, 1 mM EDTA, 0.5% Triton X-100, and cOmplete protease inhibitors), and the viral E protein quantitated by sandwich ELISA (see below). The amount of E protein detected correlates with the level of virus infectivity, which was presented as percentage of ZIKV infectivity relative to the control (i.e., virus not preincubated with immune sera). The MN_50_ titer was defined as the serum dilution that neutralized ZIKV infection by 50%.

### Sandwich ELISA to assess ZIKV infectivity.

ELISA plates were coated with 3 μg/ml of purified pan-flavivirus monoclonal antibody (MAb) D1-4G2-4-15 (ATCC HB112TM) in PBS, incubated overnight at room temperature (RT), and subsequently blocked for 2 h at RT with PBS containing 0.05% Tween 20 and 2% skimmed milk powder. After washing with PBS containing Tween 20 (PBST), ZIKV-infected cell lysates were added and incubated for 1 h at RT. Wells were washed with PBST, incubated with anti-ZIKV E polyclonal R34 IgG (9) at 6 μg/ml in PBST for 1 h at RT, and washed again. Antibodies bound to ZIKV envelope protein were detected using HRP-conjugated anti-rabbit IgG 7090 (Abcam) and TMB substrate (Life Technologies).

### Quantitation of viral RNA by RT-qPCR.

Viral RNA was extracted from 10 μl of mouse sera using a QIAamp viral RNA minikit (Qiagen) or from approximately 20 mg of organs homogenized with a Precellys lysing kit for hard tissue grinding (Bertin Technologies) and using RNeasy viral minikit. The viral load was measured by RT-qPCR using a One-Step SYBR PrimeScript RT-PCR kit II (TaKaRa). *C_T_* values for serum samples were used to calculate serum viral titer according to regression equation built by RNA extracted from 10 μl of 10^2^ to 10^6^ PFU/ml of ZIKV (PRVABC59 or MP1751). In the case of relative organ viral load, *C_T_* values of the ZIKV gene and internal control B2M gene were used for calculating Δ*C_T_* values. Average Δ*C_T_* from PBS-injected mice was used as reference to calculate ΔΔ*C_T_* values. The primer pair for the PRVABC59 ZIKV gene was 5′-GTTGTCGCTGCTGAAATGGA-3′ (forward) and 5′-GGGGACTCTGATTGGCTGTA-3′ (reverse). The primer pair for the MP1751 ZIKV gene was 5′-ACTTCCGGTGCGTTACATGA-3′ (forward) and 5′-GGGCTTCATCCATGATGTAG-3′ (reverse). The primer pair for the B2M gene was 5′-CGGCCTGTATGCTATCCAGA-3′ (forward) and 5′-GGGTGAATTCAGTGTGAGCC-3′ (reverse).

### ADE assay.

Ten-fold serial dilutions of pooled sera were mixed with 4 × 10^3^ PFU of each virus and incubated for 1.5 h at 37°C before mixing with 4 × 10^4^ K562 cells. After incubation at 37°C for 2 days (for WNV) or 3 days (all other viruses), cells were fixed with 2% paraformaldehyde (PFA) for 30 min and then washed in PBS. Blocking and permeabilization buffer (0.1% saponin, 2% FBS, 0.1% NaN_3_ in PBS) was added to cells for 30 min at 4°C. Cells are incubated with MAb 4G2 (1 μg/ml) for 1 h at 4°C followed by secondary anti-mouse Alexa Fluor 488 (Jackson ImmunoResearch, 1:50,000). After washing with PBS, cells are resuspended in blocking buffer without saponin and analyzed by cytofluorimetry in a FACSCalibur (BD Biosciences) instrument.

### Statistical analysis.

Normality was determined by Ryan-Joiner normality test with Minitab software. Statistical analysis was conducted as indicated in figure legends, with Minitab or GraphPad Prism software (*, *P* < 0.05; **, *P* < 0.01; ***, *P* < 0.001; where asterisks are missing, the differences were calculated as nonsignificant [ns]).
